# Retrospective analysis of factors associated with aortic remodeling in patients with Stanford type B aortic dissection after thoracic endovascular aortic repair

**DOI:** 10.1186/s13019-021-01571-2

**Published:** 2021-07-07

**Authors:** Biao Yu, Tangzhiming Li, Huadong Liu

**Affiliations:** 1 The Second Clinical Medical College, Jinan University, 1017 Dongmen North Road, Shenzhen, 518020 Guangdong China; 2 The First Affiliated Hospital, Southern University of Science and Technology (Department of Cardiology, Shenzhen Cardiovascular Minimally Invasive Medical Engineering Technology Research and Development Center, Shenzhen People’s Hospital), Shenzhen, 518020 Guangdong China

**Keywords:** Type B aortic dissection, Thoracic endovascular aortic repair, Aortic remodeling

## Abstract

**Objective:**

Acute aortic dissection is a life-threatening condition. Thoracic endovascular aortic repair (TEVAR), together with optimized medical treatment, is currently the first line treatment for acute Stanford type B aortic dissection. TEVAR can close the entry tear and reduce mortality. Aortic remodeling after TEVAR can directly affect the patient’s long-term prognosis. The factors that influence aortic remodeling have, however, received insufficient clinical attention and remain unclear. It is very important to identify these factors.

**Methods:**

A total of 100 patients were continuously enrolled from 2011 to 2018 in 2 centers. Relevant data, including time from hospital admission to surgery, medicine use and aortic computed tomography angiography images obtained before and 6 months after surgery were collected. Patients were divided into favorable and adverse aortic remodeling groups, according to the degree of aortic remodeling. Analysis of variance and the chi-square test were performed using SPSS software to compare differences between groups and to determine the factors that influence postoperative aortic remodeling.

**Results:**

The proportion of single-stent implantations was higher in the favorable remodeling group than in the adverse remodeling group (79.5% vs. 53.8% in distal end of stent-graft level and 81.3% vs. 56.4% in diaphragm level, respectively, *p* < 0.05). The earlier the TEVAR procedure was performed, the better the aortic remodeling (3.4 days vs. 4.8 days in distal stent graft levels, and 3.6 days vs. 4.9 days in diaphragm level, respectively, *p* < 0.05), the presence of residual distal entry tears in the abdominal aorta also improved aortic remodeling after TEVAR (85.7% vs. 55.1% in the celiac trunk level, and 92.0% vs. 48.9% in the right renal artery level, respectively, *p* < 0.05).

**Conclusion:**

Single stent-graft implantation and early surgery were associated with favorable aortic remodeling. Distal entry tears were also conducive to aortic remodeling after surgery for aortic dissection.

**Supplementary Information:**

The online version contains supplementary material available at 10.1186/s13019-021-01571-2.

## Highlights

Data, including baseline clinical parameters, complications, treatment administered during hospitalization and details of surgical procedures, were collected from multiple medical centers for 100 patients who underwent thoracic endovascular aortic repair (TEVAR) and their association with successful vascular remodeling was carefully evaluated.

Single stent-graft implantation, early surgery and distal entry tear are associated with favorable aortic remodeling.

## Introduction

Aortic dissection is a life-threatening condition caused by a tear in the inner layer of the aorta, which results in a separation of the layers of the aortic wall and subsequent formation of a true lumen and a false lumen [1]. In recent years, acute aortic dissection has become a major public health burden, with increased incidence and younger age of onset resulting in extremely high mortality and disability rates.

Based on anatomical location, aortic dissection can be classified as Stanford type A (affects the ascending aorta) or Stanford type B (begins beyond the brachiocephalic vessels). For type B aortic dissection, the in-hospital mortality is as high as 10.7% [2], with most deaths due to complications of aortic dissection [3], including lethal malperfusion, aortic insufficiency, heart failure and stroke [4]. Thoracic endovascular aortic repair (TEVAR) is the first choice of treatment for acute type B aortic dissection (ATBAD) [5, 6]. Increasing evidences suggest that TEVAR has significant advantages over open surgery in patients with ATBAD [7, 8].

Favorable vascular remodeling is the process of reducing the volume of the false cavity of the aortic dissection after TEVAR. Favorable vascular remodeling refers to a more stable structure of the vascular false cavity. Generally speaking, the greater change in CT value, the better the vascular remodeling. In contrast, adverse aortic remodeling is in opposite.

Simple TEVAR does not, however, guarantee favorable remodeling and the factors that influence long-term aortic remodeling in patients are currently not well understood. Identification of potential characters in baseline clinical characteristics or clinical treatment between patients with favorable and adverse aortic remodeling after TEVAR may play a vital role in predicting the prognosis of patients.

## Methods

### Study population

We retrospectively reviewed clinical data for 178 patients with ATBAD who underwent TEVAR in Shenzhen People’s Hospital and Guangdong Provincial People’s Hospital from January 2011 to December 2018. Aortic remodeling after TEVAR was analyzed using pre- and post-operative aortic computed tomography angiography (CTA) and 100 patients with complete data were included in the final analysis.

### Data collection and definitions

Patient data, sex, age, time of hospital admission, time from admission to surgery, medication use(β-blockers/ ACEI/ ARB/ CCB/ Aspirin), complications (Pleural effusion), presence or absence of endoleaks after surgery, and surgical methods, were collected. Endoleaks near both the entry tear and aortic stent graft were recorded in this study. Aortic bare stents are special stents that are not coated with drugs and membrane. The corresponding variable is a conventional stent covered with a membrane. Surgical methods included single stent-graft implantation (placement of a single aortic stent-graft) and complex stent-graft implantation (placement of ≥2 stent-grafts). According to guideline recommendations [9], all patients underwent CTA before and 6 months after surgery. Aortic CTA data were collected and used to assess aortic remodeling at different levels (left subclavian artery, distal edge of stent-graft, left ventricle, diaphragm, celiac trunk and right renal artery.).

### Assessment of aortic remodeling based on aortic CTA images

After aortic dissection, the shape of the true and false lumens was irregular. Measurements of the area at different levels can be converted into mean minimum and maximum diameters of the true or false lumens [10]. The change in the mean diameters of the two lumens better reflected the degree of aortic remodeling within a few months after surgery for dissection. Changes in diameter were measured at six levels: left subclavian artery, distal end of stent-graft, left ventricle, diaphragm, celiac trunk, and right renal artery . The degree of aortic remodeling was determined by the changes in the ratio of false lumen to total aortic diameter before and after surgery. The mean diameters of the true and the false lumens were measured, and the ratio of false lumen to total aortic diameter was calculated according to the following formula: mean diameter of false lumen/ mean diameter of false lumen +mean diameter of true lumen. The degree of aortic remodeling was determined according to the changes in the ratio of false lumen to total aortic diameter before and after surgery.

### Statistical analysis

Differences between patients with favorable and adverse aortic remodeling were analyzed using SPSS statistical software. Categorical variables were analyzed using the chi-square test and are presented as percentages. Continuous variables were analyzed using analysis of variance (ANOVA) and are expressed as means ± standard deviation (SD). In both cases, *p* < 0.05 was considered to be statistically significant.

## Results

**Fig. 1:**
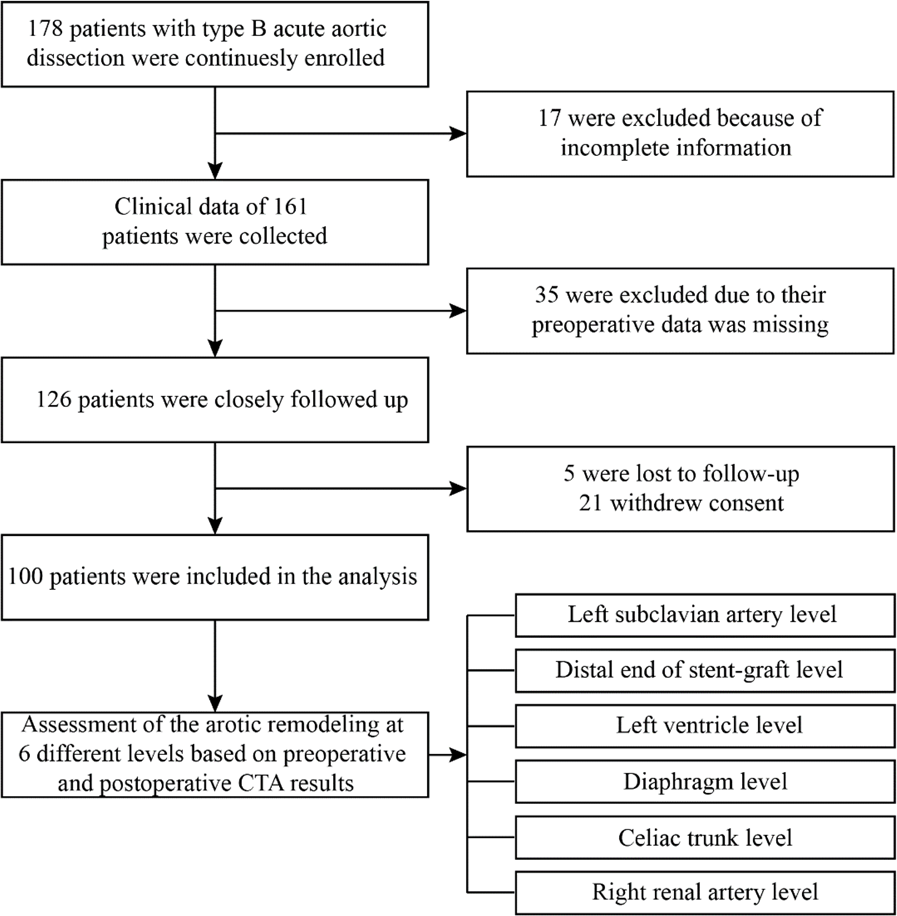
Study flow chart of included patients

178 patients with ATBAD were continuously enrolled from 2011 to 2018 (Fig. 1). The patients received optimal medicine upon hospital admission and underwent TEVAR when their basal blood pressure was controlled within a reasonable range (systolic blood pressure 100–120 mmHg). All patients were followed up for at least 6 months. 100 patients were included in the final analysis.

No significant differences were found in medicine use (angiotensin converting enzyme inhibitors, angiotensin receptor blockers, β-blockers, calcium channel blockers, and aspirin), the presence or absence of pleural effusion or D-dimer levels between the favorable and unfavorable aortic remodeling groups (*p* > 0.05) (supplemental files).

CTA images acquired before and 6 months after surgery were used to evaluate the degree of aortic remodeling. Among 100 patients with ATBAD, six different levels were chosen for sections: left subclavian artery, distal end of stent-graft, left ventricle, diaphragm, celiac trunk and right renal artery. The cross-sectional diameters of the true and false lumens at the six levels were calculated, and the ratio of false lumen to total aortic diameter was then calculated.

**Fig. 2:**
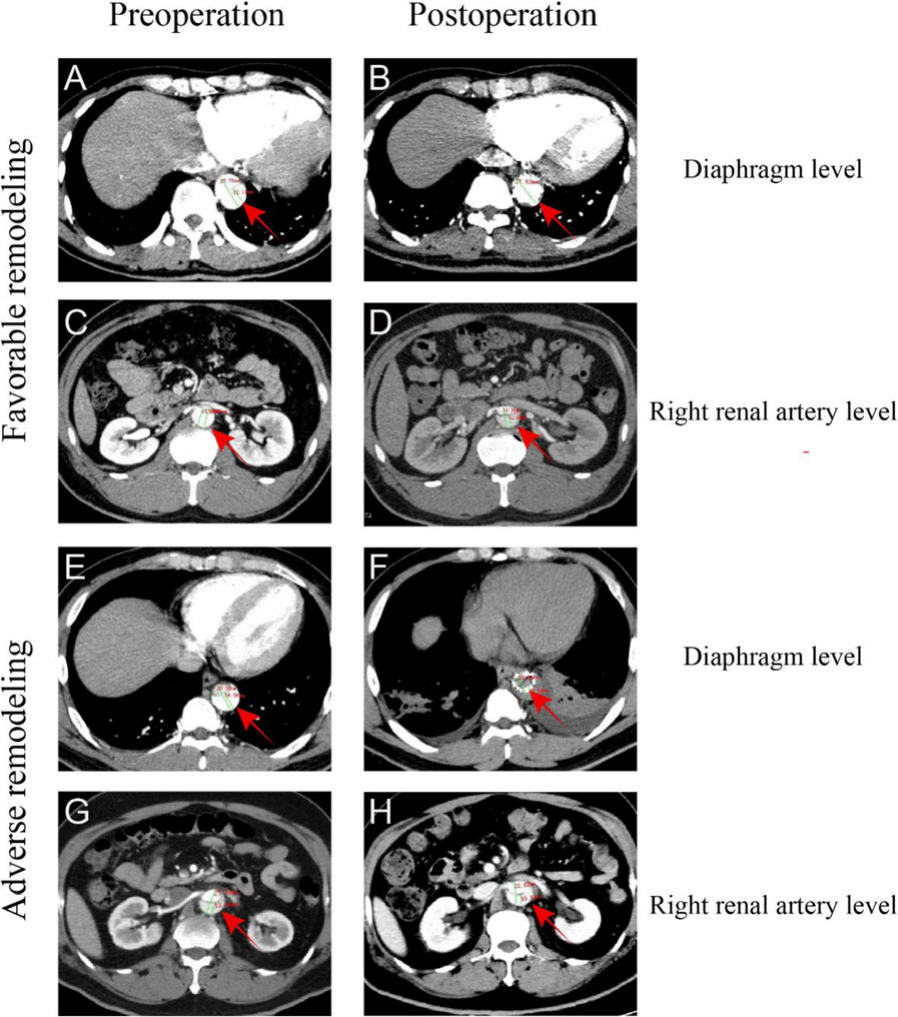
CT images, before surgery and after TEVAR, showing favorable and adverse aortic remodeling. A and B: Favorable aortic remodeling before and after surgery observed at the diaphragm level. The false lumen had almost disappeared. C and D: Favorable aortic remodeling before and after surgery observed at the level of the right renal artery. The false lumen had almost disappeared. E and F: Adverse aortic remodeling before and after surgery observed at the diaphragm level. The false lumen obviously enlarged. G and H: Adverse aortic remodeling before and after surgery observed at the level of the right renal artery. The false lumen had no obviously change after 6 months. Red arrows indicate the location of dissected lesions

CTA images before and 6 months after surgery indicated a significant difference in healing of the false lumen between patients with favorable and adverse aortic remodeling. In patients with favorable aortic remodeling at the diaphragm level, the false lumen had almost disappeared 6 months after surgery (Fig. 2A and B) and outcomes were significantly improved. Poor postoperative aortic remodeling at the diaphragm level was associated with obviously enlarged hematoma and slightly increased ipsilateral pleural effusions (Fig. 2E and F). The postoperative recovery of these patients was poor and they required close follow up, with control of blood pressure and heart rate, to prevent serious vascular complications.

Identification of factors associated with favorable aortic remodeling is important to evaluate the long-term prognosis of patients. We further analyzed differences in baseline clinical data and surgical conditions between patients with aortic dissection at the six different levels who had favorable and adverse aortic remodeling. Because of differences in entry tears and artery involvement, the effective number of patients with aortic dissections at the levels of the left subclavian artery, the distal end of the stent-graft, the left ventricle, the diaphragm, the celiac trunk and the right renal artery were 12, 78, 95, 96, 99 and 96, respectively.

Aortic remodeling after surgery was found to be closely associated with time from hospital admission to surgery, surgical method and patency of distal entry tears. As shown in Table 1, at the levels of the distal end of the stent-graft and the diaphragm, a higher proportion of patients who received single-stent implantation had favorable aortic remodeling after surgery, compared with those who received complex stent-graft implantation (79.5% vs. 53.8%, *p* < 0.05 and 81.3% vs. 56.3%, respectively, *p* < 0.05). Patients who underwent surgery soon after admission also had better aortic remodeling than those who underwent late surgery (3.4 ± 2.5 vs. 4.8 ± 3.4, *p* < 0.05 and 3.6 ± 2.6 vs. 4.9 ± 3.2, *p* < 0.05). At the levels of the celiac trunk and right renal artery, better remodeling with present distal tears (85.7% vs. 55.1 and 92.0% vs. 48.9%, respectively, *p* < 0.05). The association between endoleaks and aortic remodeling was only analyzed at the levels of the subclavian artery and the distal end of the stent-graft because endoleaks only affected the proximal stent attachment site. There was no significant association between endoleaks and aortic remodeling at the level of the distal end of the stent-graft (12.8% vs. 10.3%, *p* > 0.05).

**Table 1:** Data showing influencing factors between groups at difference levels

Level of aortic dissection	Variable	Total number	Favorable aortic remodeling	adverse aortic remodeling	*p* value
**Left subclavian artery Level (**total *n* = 12, *n* = 6 in each group)	Aortic bare stent placement (n (%))	0 (0%)	0 (0%)	0 (0%)	1
	Endoleak (n (%))	0 (0%)	0 (0%)	0 (0%)	1
	Surgical method (placement of one single stent-graft, n (%))	7 (58.3%)	4 (66.7%)	3 (50%)	0.558
	Time from admission to surgery (mean days ± SD)	5.3 ± 3.6	4.8 ± 3.7	5.7 ± 8	0.708
**Distal end of stent-graft Level (**total *n* = 79, *n* = 39 in each group)	Aortic bare stent placement (n (%))	11 (14.1%)	5 (12.8%)	6 (15.4%)	0.745
	Endoleak (n (%))	10 (12.8%)	4 (10.3%)	6 (15.4%)	0.498
	Surgical method (placement of single stent graft, n (%))	52 (66.7%)	31 (79.5%)	21 (53.8%)	0.016
	Time from admission to surgery (mean ± SD)	4.1 ± 3.0	3.4 ± 2.5	4.8 ± 3.4	0.028
**Left ventricle Level (**total *n* = 95, *n* = 48 and 47 in each group)	Aortic bare stent placement (n (%))	11 (11.6%)	6 (12.5%)	5 (10.6%)	0.777
	Surgical method (placement of single stent graft, n (%))	65 (68.4%)	34 (70.8%)	31 (66%)	0.609
	Time from admission to surgery (mean ± SD)	4.2 ± 3.0	4.4 ± 3.1	4.0 ± 2.8	0.587
**Diaphragm Level (**total *n* = 96, *n* = 48 in each group)	Aortic bare stent placement (n (%))	11 (11.5%)	6 (12.5%)	5 (10.4%)	0.749
	Surgical method (placement of single sent graft, n (%))	66 (68.8%)	39 (81.3%)	27 (56.3%)	0.008
	Time from admission to surgery (mean ± SD)	4.2 ± 2.9	3.6 ± 2.6	4.9 ± 3.2	0.031
**Celiac trunk Level (**total *n* = 99, *n* = 50 and 49 in each group)	Aortic bare stent placement (n (%))	11 (11.1%)	6 (12%)	5 (10.2%)	0.776
	Surgical method (placement of single sent graft, n (%))	66 (66.7%)	32 (64%)	34 (69.4%)	0.570
	Time from admission to surgery (mean ± SD)	4.3 ± 3.0	4.4 ± 3.0	4.2 ± 3.0	0.720
	Distal entry tears (presence, n (%))	69 (70.4%)	42 (85.7%)	27 (55.1%)	0.000
**Right renal artery Level (**total *n* = 96, *n* = 48 in each group)	Aortic bare stent placement (n (%))	11 (11.5%)	8 (16.7%)	3 (6.3%)	0.109
	Surgical methods (placement of single sent graft, n (%))	64 (66.7%)	34 (70.8%)	30 (62.5%)	0.386
	Time from admission to surgery (mean ± SD)	4.3 ± 3.0	4.5 ± 3.2	4.1 ± 2.9	0.546
	Distal entry tears (presence, n (%))	69 (71.1%)	46 (92.0%)	23 (48.9%)	0.000

## Discussion

Our study provided further clinical evidence that strengthens guidance for the management of patients during hospitalization and showed that performing surgery as soon as possible promoted better aortic remodeling. We found that single stent-graft implantation and distal entry tear are associated with favorable aortic remodeling.

Favorable aortic remodeling significantly improves the prognosis of patients with aortic dissection after surgery. Previous studies showed that the 3-year mortality of patients with an enlarged vascular lumen caused by insufficient thrombosis of the false lumen and poor vascular remodeling after TEVAR tends to be higher [11]. This means that the degree of false lumen thrombosis and vascular remodeling will directly affect the prognosis of patients with aortic dissection after surgery [12, 13]. A previous study, which focused more on investigating aortic remodeling in patients with acute and chronic aortic dissection after TEVAR, showed that TEVAR cannot help to improve aortic remodeling in patients with chronic aortic dissection [14]. There have, however, been very few reports describing the differences in surgical effect and prognosis of patients with acute aortic dissection who underwent TEVAR, leading to a lack of guidance for the management of patients with ATBAD who were hospitalized for stent graft-implantation.

In this study, there was no significant difference in baseline data, including age, blood pressure, medication use, endoleaks and Aortic bare stent implements between patients in the favorable and adverse aortic remodeling groups, indicating good consistency between the two groups.

We found that one single stent-graft implantation and a shorter time from hospital admission to the TEVAR procedure were associated with a good prognosis. We believe that this may because of good vascular condition and better control of risk factors, such as hypertension, have a positive effect on favorable aortic remodeling. It is generally believed that this type of patient has a relatively simple condition, fewer complications and better postoperative prognosis. Following hospital admission, early surgery for patients with aortic dissection can promote favorable aortic remodeling. Closure of the entry tears may lead to rapid healing and remodeling of the dissected aorta [1]. When surgery is delayed, the vascular lesion worsens and it may be difficult to completely cover the false lumen [15]. Our findings are consistent with previous studies [16, 17], which showed that delayed surgery has an adverse effect on the prognosis of patients with acute aortic dissection after maintaining a stable internal environment by administering adequate medical treatment [2].

Performing TEVAR during the acute phase of aortic dissection can promptly seal off entry tears and prevent blood flow into the false lumen, leading to thrombosis of the false lumen and thereby enlarging the true lumen and stimulating aortic remodeling [18]. Based on optimized medical treatment, performing TEVAR as early as possible can, therefore, prevent blood flow into the aortic wall, enlarge the true lumen and mitigate poor dynamic blood perfusion of the viscera and lower extremities. On the other hand, collapse of the false lumen avoids insufficient thrombosis caused by continuous impingement of blood flow on the false lumen and ultimately affects vascular remodeling after the operation. With no blood flow at the distal end of the false cavity, the organization of the thrombus begins to appear within 1 or 2 days [19].

Distal aortic remodeling is controlled by blood flow between the true and false lumens as well as by pressure in the false lumen. High pressure in the false lumen can lead to poor aortic remodeling [20]. In patients with aortic dissection, if the intimal tear is confined to the thoracic aorta and there is no patent entry tear in the abdominal aortic segment, a false lumen would not exist in the abdominal aortic segments after the entry tear of the dissection is completely covered by a stent-graft. However, if the entry tear is not completely covered by the stent-graft and there is also no patent entry tear in the abdominal aorta, the pressure in the false lumen would be higher than in those with a patent distal tear after covering the proximal intimal tear [21]. This can lead to incomplete thrombosis in the false lumen, thereby affecting remodeling in distal aorta. Aortic remodeling of the abdominal aorta is, therefore, associated with the presence or absence of a patent entry tear in the abdominal aorta. To achieve better postoperative aortic remodeling in patients who do not have distal entry tears, it is necessary to strictly control blood pressure before the stent-graft implantation and to regularly follow-up by CT examination to observe the remodeling of the abdominal aorta after surgery.

In this study, we found that endoleak after TEVAR had little effect on aortic remodeling. This may be because of the low incidence of endoleak in patients included in this study (only 1%). Endoleak can increase blood flow in the false lumen, thus increasing the pressure inside the false lumen and slowing remodeling of the aorta. Although endoleak is a surgical adverse event that should be avoided, most endoleaks are self-limiting. Only severe endoleaks impinge on blood flow in the proximal blood vessels, which aggravates the patient’s condition [22] and can even cause traumatic thoracic aortic injury [23]. Endoleaks thus have no obvious guiding significance for the management of patients with ATBAD.

This study had some limitations that should be borne in mind. Because it was a retrospective study, we cannot establish a cohort for specific interventions. The number of patients enrolled in the group was limited. we need to boost more credibility in further research. The incidence of endoleaks was also low, so the factors affecting aortic remodeling after surgery in patients with ATBAD could not be fully elucidated. Further prospective studies with larger sample sizes are needed to confirm the relationship between the time of operation, aortic remodeling and the prognosis of patients with ATBAD after surgery.

## Conclusions

Our study had four main findings. 1) For patients with uncomplicated acute aortic dissection, the entry tears could be closed by placing a single stent-graft, and these patients were more likely to have better aortic remodeling. 2) Once the patient’s blood pressure was reduced below the normal range by optimal medical treatment, the earliest possible TEVAR resulted in more complete thrombosis formation in the false lumen and better aortic remodeling. 3) In the distal end of aortic dissection which was not covered by the stent graft, aortic remodeling was associated with the presence or absence of a patent distal entry tear. It was, therefore, important that patients without a distal entry tear received antihypertensive treatment before surgery.

## Supplementary Information

**Additional file 1.**

## Data Availability

The datasets used or analysed during the current study are available from the corresponding author on reasonable request.

## Abbreviations

TEVAR: Thoracic endovascular aortic repair
ATBAD: Acute Stanford type B aortic dissection
CTA: Computed tomography angiography
ANOVA: Analysis of variance
SD: Standard deviation
