# Supplementary material for: Retrospective analysis of factors associated with aortic remodeling in patients with Stanford type B aortic dissection after thoracic endovascular aortic repair
Source: J Cardiothorac Surg. 2021 Jul 7;16:190. doi: 10.1186/s13019-021-01571-2 (PMC8262045; doi:10.1186/s13019-021-01571-2)
Supplement: Supplementary file 1 — Additional file 1. [file 13019_2021_1571_MOESM1_ESM.docx]

Table 1 Baseline characteristics of patients with type B aortic dissection who obtained favorable and adverse aortic remodeling before and after TEVAR at the level of the left subclavian artery (n=12)

| Variables | Total number | Favorable aortic remodeling (n=6) | Adverse aortic remodeling (n=6) | P value |
| --- | --- | --- | --- | --- |
| Sex (male, n/%) | 10（83.3%） | 5（83.3%） | 5（83.3%） | 1.000 |
| Age (means ± SD) | 51.3±11.8 | 54.5±14.3 | 48.0±8.6 | 0.363 |
| Hypertension (n/%) | 10（83.3%） | 6（100%） | 4（66.7%） | 0.439 |
| Pleural effusion (n/%) | 1（8.3%） | 1（16.7%） | 0（0%） | 1.000 |
| β-blockers (n/%) | 11（91.7%） | 6（100%） | 5（83.3%） | 1.000 |
| ACEI (n/%) | 6（50%） | 3（50%） | 3（50%） | 1.000 |
| ARB (n/%) | 0（0%） | 0（0%） | 0（0%） | - |
| CCB (n/%) | 10（83.3%） | 5（83.3%） | 5（83.3%） | 1.000 |
| Aspirin (n/%) | 0（0%） | 0（0%） | 0（0%） | - |
| D-Dimer levels (means ± SD) | 5797.6±3878.1 | 5638.6±4839.9 | 5861.2±4084.8 | 0.952 |

Table 2 Baseline characteristics of patients with type B aortic dissection who obtained favorable and adverse aortic remodeling before and after TEVAR at the level of the distal end of the stent graft (n=78)

| Variables | Total number | Favorable aortic remodeling (n=39) | Adverse aortic remodeling (n=39) | P value |
| --- | --- | --- | --- | --- |
| Sex (male, n/%) | 71（91.0%） | 34（87.2%） | 37（94.9%） | 0.428 |
| Age (means ± SD) | 52.7±10.6 | 53.1±9.9 | 52.3±11.3 | 0.726 |
| Hypertension (n/%) | 62（79.5%） | 30（76.9%） | 32（81.2%） | 0.575 |
| Pleural effusion (n/%) | 39（50.0%） | 19（48.7%） | 20（51.3%） | 0.821 |
| β-blockers (n/%) | 76（97.4%） | 38（97.4%） | 38（97.4%） | 1.000 |
| ACEI (n/%) | 15（19.2%） | 10（25.6%） | 5（12.8%） | 0.151 |
| ARB (n/%) | 28（35.9%） | 15（38.5%) | 13（33.3%） | 0.637 |
| CCB (n/%) | 56(71.8%) | 28(71.8%) | 28(71.8%) | 1.000 |
| Aspirin (n/%) | 2(2.6%) | 0(0%) | 2(5.1%) | 0.474 |
| D-Dimer levels (means ± SD) | 5041.5±5717.3 | 5101.3±1002.6 | 4981.6±943.4 | 0.931 |

Table 3 Baseline characteristics of patients with type B aortic dissection who obtained favorable and adverse aortic remodeling before and after TEVAR at the level of the left ventricle (n=95)

| Variables | Total number | Favorable aortic remodeling (n=48) | Adverse aortic remodeling (n=47) | P value |
| --- | --- | --- | --- | --- |
| Sex (male, n/%) | 86(90.5%) | 42(87.5%) | 44(93.6%) | 0.504 |
| Age (means ± SD) | 52.5±10.5 | 54.0±10.0 | 51.0±11.4 | 0.177 |
| Hypertension (n/%) | 78(82.1%) | 40(83.3%) | 38(80.9%) | 0.752 |
| Pleural effusion (n/%) | 44(46.3%) | 25(52.1%) | 19(40.4%) | 0.255 |
| β-blockers (n/%) | 92(96.8%) | 46(95.8%) | 46(97.9%) | 1.000 |
| ACEI (n/%) | 27(28.4%) | 12(25%) | 15(31.9%) | 0.455 |
| ARB (n/%) | 28(29.5%) | 18(37.5%) | 10(21.3%) | 0.083 |
| CCB (n/%) | 70(73.7%) | 35(72.9%) | 35(74.5%) | 0.864 |
| Aspirin (n/%) | 2(2.1%) | 2(4.2%) | 0(0%) | 0.096 |
| D-Dimer levels (means ± SD) | 4871.9±5485.3 | 4620.9±5481.4 | 5244.9±5560.0 | 0.627 |

Table 4 Baseline characteristics of patients with type B aortic dissection who obtained favorable and adverse aortic remodeling before and after TEVAR at the level of the diaphragm (n=96)

| Variables | Total number | Favorable aortic remodeling (n=48) | Adverse aortic remodeling (n=48) | P value |
| --- | --- | --- | --- | --- |
| Sex (male, n/%) | 87(90.6%) | 43(89.6%) | 44(91.7%) | 1.000 |
| Age (means ± SD) | 52.3±10.9 | 53.2±10.2 | 51.4±11.6 | 0.418 |
| Hypertension (n/%) | 79(82.3%) | 37(77.1%) | 42(87.5%) | 0.181 |
| Pleural effusion (n/%) | 45(46.9%) | 26(54.2%) | 19(39.6%) | 0.152 |
| β-blockers (n/%) | 93(96.9%) | 46(95.8%) | 47(97.9%) | 1.000 |
| ACEI (n/%) | 28(29.2%) | 14(29.2%) | 14(29.2%) | 1.000 |
| ARB (n/%) | 28(29.2%) | 16(33.3%) | 12(25%) | 0.369 |
| CCB (n/%) | 71(70.4%) | 35(72.9%) | 36(75%) | 0.816 |
| Aspirin (n/%) | 2(2.1%) | 1(2.1%) | 1(2.1%) | 1.000 |
| D-Dimer levels (means ± SD) | 4826.5±5464.3 | 5180.5±6000.2 | 4413.4±4815.5 | 0.540 |

Table 5 Baseline characteristics of patients with type B aortic dissection who obtained favorable and adverse aortic remodeling before and after TEVAR at the level of the celiac trunk (n=99)

| Variables | Total number | Favorable aortic remodeling (n=50) | Adverse aortic remodeling (n=49) | P value |
| --- | --- | --- | --- | --- |
| Sex (male, n/%) | 90(90.9%) | 45(90%) | 45(91.8%) | 1.000 |
| Age (means ± SD) | 50.1±10.7 | 53.9±11.4 | 50.2±9.7 | 0.085 |
| Hypertension (n/%) | 82(82.8%) | 42(84%) | 40(81.6%) | 0.755 |
| Pleural effusion (n/%) | 45(45.5%) | 23(46%) | 22(44.9%) | 0.912 |
| β-blockers (n/%) | 96(97.0%) | 49(98%) | 47(95.9%) | 0.986 |
| ACEI (n/%) | 29(29.3%) | 13(26%) | 16(32.7%) | 0.467 |
| ARB (n/%) | 28(28.3%) | 13(26%) | 15(30.6%) | 0.610 |
| CCB (n/%) | 74(74.7%) | 36(72%) | 38(77.6%) | 0.525 |
| Aspirin (n/%) | 2(2%) | 1(2%) | 1(2%) | 1.000 |
| D-Dimer levels (means ± SD) | 4860.8±5412.9 | 4812.4±5985.7 | 4920.0±4702.1 | 0.930 |

Table 6 Baseline characteristics of patients with type B aortic dissection who obtained favorable and adverse aortic remodeling before and after TEVAR at the level of the right renal artery (n=96)

| Variables | Total number | Favorable aortic remodeling (n=48) | Adverse aortic remodeling (n=48) | P value |
| --- | --- | --- | --- | --- |
| Sex (male, n/%) | 87(90.6%) | 43(89.6%) | 44(91.7%) | 0.726 |
| Age (means ± SD) | 51.8±10.5 | 54.5±10.0 | 49.2±10.5 | 0.012 |
| Hypertension (n/%) | 79(82.3%) | 38(79.2%) | 41(85.4%) | 0.423 |
| Pleural effusion (n/%) | 44(45.8%) | 22(45.8%) | 22(45.8%) | 1.000 |
| β-blockers (n/%) | 93(96.9%) | 46(95.8%) | 47(97.9%) | 1.000 |
| ACEI (n/%) | 27(28.1%) | 11(22.9%) | 16(33.3%) | 0.256 |
| ARB (n/%) | 28(29.2%) | 14(29.2%) | 14(29.2%) | 1.000 |
| CCB (n/%) | 72(75.0%) | 34(70.8%) | 38(79.2%) | 0.346 |
| Aspirin (n/%) | 2(2.1%) | 2(4.2%) | 0(0%) | 0.475 |
| D-Dimer levels (means ± SD) | 4978.7±5483.8 | 5636.4±6497.5 | 4189.5±3890.3 | 0.252 |
